# Recent Observations in Immunity Production

**Published:** 1920-09-11

**Authors:** 


### A NEW FACTOR IN TREATMENT.

### Recent Observations in Immunity Production.

It is perhaps early to draw definite conclusions from the new conceptions of the mechanism and possibilities of vaccine treatment, as recently published in the *Annales* of the Pasteur Institute of Paris, but in agreement with the *British Medical Journal*, we must admit that to all appearances they are "of such enormous practical importance and so subversive of the ideas hitherto held by bacteriologists that they merit close study by the whole of the medical profession." Unfortunately, as with most modern publications on technical subjects, it is by no means easy for the majority of the unspecialised at once to understand the true import of the new observations, and it is our aim, therefore, briefly to outline the main points wherein the novelty of these reported discoveries lies.

### Dr. Besredka's Tests.

In the past we have always taken as the first necessity in combating bacterial disease urgency in preventing the passage of the infection throughout the system, but let us now follow the earlier experiments described. In attempting to produce immunity against bacillary dysentery (Shiga), Dr. Besredka injected living germs into the veins of rabbits. These animals died of dysentery, but *post-mortem* it was found that the activity of the germs and the germs themselves were localised to the intestines, just as in the course of the usual human alimentary infection. The micro-organisms circulating in the blood appeared to lose no time in localising themselves, and did not attempt to produce an increasing septicæmia—in fact, their presence in the blood is described as apparently harmless. Here, therefore, we see a complete reversal of the idea of forcing the organisms out of the circulation by the production of immune substances in the blood stream. Other methods of inserting the bacilli into the system were tried—subcutaneous, intracutaneous—but in every case the organisms apparently travelled direct to the intestinal mucosa before producing any serious harmful effects. Only when they reached this particular area did acute disease begin.

The question now arose as to what would happen when *dead* bacilli were similarly injected. What anti-bodies are produced and where? Experiments, too detailed and complex to be described here, appear to indicate that no anti-bodies are produced in the blood stream itself, which fluid seems to play an entirely secondary process in any immunity gained. The animal itself is, however, protected to a high degree, and this power appears to lie entirely in the intestinal wall—*i.e.*, in "an intestinal barrier." The blood of animals previously inoculated with dead germs—*i.e.*, vaccines—shows none of our accepted anti-body reactions, and yet these animals cannot be infected by later ingestion of living organisms. In other words, they have an immunity, high but entirely localised in nature.

### The Intestinal Barrier.

The protection is, therefore, not by circulating immune substances, but by an intestinal barrier strengthened against the effects of the micro-organisms; the connection between this fact and the curious natural immunity of rabbits and other small animals to typhoid infections was at once realised by the Paris workers. The intestines of these mammals apparently oppose naturally an "intestinal barrier" to typhoid, and yet they have no natural anti-bodies in their blood. What is the nature of the local protection? Does the barrier correspond to that artificially produced by inoculating with dysentery vaccine? Is there any way in which we can break down this barrier experimentally?

An answer was quickly forthcoming in that previous administration of ox bile makes rabbits immediately susceptible to the typhoid group of organisms, which at once attacks the tissues in the customary intestinal sites. In brief, a normal rabbit given living typhoid germs by the mouth shows no ill-effects, and when infected intravenously shows pathological changes only when very large doses are given. On the other hand, a rabbit previously fed on bile always develops the disease when infected via the alimentary canal, and requires a very small intravenous dose to produce death.

Between these experimental results a clear parallel is drawn by a correspondent in the *Times*, who makes the following summary, and one which should be closely followed:

(1) Besredka was able to make rabbits (which are not normally immune) immune to dysentery by raising an intestinal or local barrier. This was achieved by giving them dead germs to eat (preferably mixed with bile). The animals could "bear with impunity an inoculation of living virus so severe that it kills the untreated controls, rabbits or mice, within twenty-four hours."

(2) He was able to make rabbits (which are normally immune) liable to typhoid and paratyphoid by lowering their intestinal or local barrier. This was achieved by giving them ox bile to eat. These animals then readily contracted typical disease when they took typhoid or paratyphoid germs by mouth.

The practical points which these results imply are: (*a*) That against certain types of bacterial infection a distinct and definite local barrier of immunity may be set up, and that in the case of these intestinal infections we have ascertained one method of destroying it, and, more important, a method of producing it artificially, *i.e.*, by the *oral* administration of the corresponding vaccines—the dead bacilli. (*b*) That the cause, or partial cause, of human infection may be due to simple bodily derangements, comparable to the feeding of the experimental animals with bile. In any case, they distinctly point to the fact that we have to appreciate their power in producing disease, not only to

**A New Factor in Treatment**——(*continued*).

examine, classify, and reclassify bacteria themselves, and the way they produce it, but we must realise also that changes in the living soil upon which they grow may be even more potent in releasing them to play havoc within the body than any intrinsic changes in the germs themselves. In any case, we have at once a new indication in the methods of vaccine therapy, and a prospect of much new light on the immediate causes for onset of bacterial activity in previously healthy tissues. We have for a long time accepted the rule that bacteria would readily attack tissues the resistance of which was lowered by cold, trauma, or pathological states; but Dr. Besredka's experiments suggest that there is much more definite information than this to be gained from laboratory investigation, and that he has made a valuable first step.

